# Metagenome-assembled genomes of 72 *Escherichia coli* strains from vaginal samples collected from pregnant women

**DOI:** 10.1128/mra.00831-24

**Published:** 2024-11-11

**Authors:** Nassim Boutouchent, Thi Ngoc Anh Vu, Luce Landraud, Sean P. Kennedy

**Affiliations:** 1 Département de Biologie Computationnelle, Institut Pasteur, Université Paris Cité, Paris, France; 2 Département de Microbiologie, CHU de Rouen, Rouen, France; 3 VNU-Institute of Microbiology and Biotechnology, Vietnam National University, Hanoi, Vietnam; 4 Université Paris Cité and Université Sorbonne Paris Nord, INSERM, IAME, Paris, France; Department of Biological Sciences, Wellesley College, Wellesley, Massachusetts, USA

**Keywords:** metagenomics, *Escherichia coli*, vaginal microbiota

## Abstract

We report 72 metagenome-assembled genomes (MAGs) of *Escherichia coli* recovered from the vaginal microbiomes of pregnant women. The MAGs have an estimated median genome size of 5.1 Mb (IQR 4.9–5.3). The average number of unique genes identified per genome was 3,330 genes (IQR 3,356–3,501), with a mean of 1,708 per genome being annotated.

## ANNOUNCEMENT

*Escherichia coli* is a common commensal bacterium of the human gut microbiota, yet many strains display pathogenic capabilities (1). *E. coli* is the predominant causative agent of urogenital infections during pregnancy as well as newborn infections (2, 3). Given its clinical significance in the vaginal compartment during pregnancy, we report 72 genomes assembled from metagenomic sequencing of vaginal swab samples from 71 distinct women during their third trimester of pregnancy.

Vaginal swabs were collected with E-swab collect tubes containing 1 mL of AIMES conservation solution, as detailed in Baud et al. (4). Automated extraction of DNA from 750 µL swab medium was performed using the QIAsymphony DSP Virus/Pathogen Kit and the Complex400_OBL protocol. Metagenomic libraries were prepared using the TruSeq kits (Illumina, San Diego, CA, USA) with a minimum of 50 ng input DNA. Average insert size >300 bp was confirmed by Agilent 2100, and qPCR was used to confirm a library concentration of >3 nM. Libraries were sequenced on a NovaSeq6000 instrument. For each sample, an average of 20 × 10^6^ paired-end reads (2 × 150 bp) with mean Phred scores > *Q*30 was generated. Human host reads were removed by mapping to the human genome (hg38). Host-free reads were taxonomically assigned using Kraken2 (5–7). Samples containing at least 30,000 reads corresponding to *E. coli* were targeted for filtering and genomic assembly through an in-house Snakemake (version 8.11.6) pipeline. *E. coli* reads were selected by Bowtie2 (version 2.5.1) mapping of host-free reads to a representative custom database of 32 RefSeq strains, which included 2 reference genomes and 30 clinical isolates with complete assembly and full genome representation (8). Read pairs with at least one reference-aligned pair were retained and assembled using SPAdes (version 3.15.5) (9). The assembled genomes were annotated using Prokka (version 1.14.5) (10). Quality markers were evaluated using an in-house Python script to generate a CSV file with detailed assembly statistics. The number of contigs for each genome assembly, along with estimates of genome completeness and contamination, were assessed using CheckM (version 1.2.3) (11). Default parameters were used for all software unless otherwise specified.

Among the 1,950 samples analyzed, 72 *E. coli* genomes were successfully assembled. The genome sizes of the assemblies had a median of 5.1 Mb (IQR 4.9–5.3 Mb). Notably, seven metagenome-assembled genomes were under 2 Mb (Table 1). The *N*_50_ values had a mean of 80 kb (SD ± 69 kb), ranging from 514 to 231.1 kb. The *N*_90_ values show a mean length of 14.8 kb (SD ± 16.7 kb). The median number of unique genes identified per genome was 3,330 genes (IQR 3,356–3,501), with a mean of 1,708 per genome being annotated.

**TABLE 1:** Accession numbers and statistics of 72 metagenomic-assembled *E. coli* genomes

Sample accession	Assembly name	Assembly accession	No. reads	Genome size (bp)	Coverage (×)	No. contigs	*N*_50_	Completeness (%)	Contamination (%)
ERS20601047	EC_IMVM_0001	GCA_964204325	1,839,507	4,484,846	123.0	234	24,125	99.02	0.18
ERS20601069	EC_IMVM_0023	GCA_964204005	3,841,745	7,428,842	155.1	4,038	695	99.50	47.31
ERS20601077	EC_IMVM_0031	GCA_964204145	7,240,947	5,007,326	433.8	411	16,806	99.64	0.57
ERS20601084	EC_IMVM_0038	GCA_964204065	7,341,650	5,109,218	431.1	314	25,975	99.64	0.22
ERS20601139	EC_IMVM_0095	GCA_964204075	7,108,646	4,973,432	428.8	274	28.838	99.64	0.22
ERS20601159	EC_IMVM_0115	GCA_964203995	15,011,489	6,045,110	745.0	3,665	513	99.16	12.82
ERS20601191	EC_IMVM_0147	GCA_964204135	426,045	4,990,410	25.6	408	11,352	99.12	0.19
ERS20601210	EC_IMVM_0166	GCA_964204235	1,002,009	1,337,948	224.7	1,775	349	38.57	6.65
ERS20601252	EC_IMVM_0208	GCA_964204165	7,346,113	5,072,383	434.5	200	45,948	99.64	0.35
ERS20601270	EC_IMVM_0227	GCA_964204195	162,866	4,749,120	10.3	717	4,559	99.02	0.99
ERS20601286	EC_IMVM_0243	GCA_964204205	703,238	5,086,506	41.5	321	37,635	99.64	0.63
ERS20601307	EC_IMVM_0265	GCA_964204185	17,527,092	5,046,522	1,041.9	275	29,819	99.64	0.89
ERS20601317	EC_IMVM_0275	GCA_964204115	1,450,739	5,711,705	76.2	1,380	2,579	99.64	16.75
ERS20601322	EC_IMVM_0280	GCA_964203975	1,875,298	5,180,179	108.6	486	13,065	99.64	0.49
ERS20601324	EC_IMVM_0282	GCA_964203945	967,824	5,482,168	53.0	1,799	1,413	99.64	17.34
ERS20601333	EC_IMVM_0291	GCA_964204035	2,470,623	5,115,675	144.9	324	27,532	99.64	0.65
ERS20601403	EC_IMVM_0362	GCA_964204095	6,774,894	4,539,097	447.8	214	23,372	99.02	0.18
ERS20601484	EC_IMVM_0446	GCA_964204125	2,755,393	5,251,807	157.4	543	12,483	99.64	0.96
ERS20601569	EC_IMVM_0531	GCA_964204255	622,881	5,390,366	34.7	1,625	2,574	99.33	5.33
ERS20601625	EC_IMVM_0587	GCA_964204215	808,892	2,567,544	94.5	5,276	263	45.49	31.78
ERS20601626	EC_IMVM_0588	GCA_964203955	6,391,304	7,757,220	247.2	7,721	401	95.93	67.57
ERS20601634	EC_IMVM_0596	GCA_964203905	7,193,337	5,066,276	426.0	313	30,471	99.64	0.51
ERS20601636	EC_IMVM_0598	GCA_964204025	820,028	5,022,299	49.0	358	27,718	99.64	0.49
ERS20601679	EC_IMVM_0641	GCA_964204245	100,655	4,541,349	6.6	1,794	993	95.84	5.10
ERS20601748	EC_IMVM_0710	GCA_964203915	1,150,832	7,061,172	48.9	10,227	202	99.42	63.83
ERS20601788	EC_IMVM_0751	GCA_964204625	938,913	1,485,538	189.6	3,038	258	31.54	3.16
ERS20601877	EC_IMVM_0840	GCA_964204425	142,943	4,720,624	9.1	1,077	2,379	98.86	3.01
ERS20601910	EC_IMVM_0873	GCA_964203985	332,916	4,935,099	20.2	309	22,488	99.64	0.38
ERS20601915	EC_IMVM_0878	GCA_964204535	6,823,049	4,839,165	423.0	140	41,503	99.64	0.73
ERS20601993	EC_IMVM_0956	GCA_964204525	49,297	5,086,315	2.9	379	9,781	99.58	1.32
ERS20602075	EC_IMVM_1038	GCA_964204055	835,263	5,659,579	44.3	1,779	1,553	99.33	16.98
ERS20602081	EC_IMVM_1044	GCA_964204225	653,862	4,899,490	40.0	253	34,686	99.64	0.26
ERS20602091	EC_IMVM_1054	GCA_964204155	1,353,699	1,191,467	340.8	1,868	294	38.27	2.48
ERS20602102	EC_IMVM_1065	GCA_964203935	1,196,420	1,423,858	252.1	1,539	442	35.96	10.53
ERS20602114	EC_IMVM_1077	GCA_964204265	4,601,258	5,089,632	271.2	109	92,775	99.64	0.22
ERS20602120	EC_IMVM_1083	GCA_964204175	371,450	4,575,903	24.4	147	45,586	99.64	0.18
ERS20602154	EC_IMVM_1117	GCA_964204085	953,634	4,949,165	57.8	433	24,555	99.64	0.18
ERS20602168	EC_IMVM_1131	GCA_964204445	5,827,066	11,093,269	157.6	28,109	133	95.45	94.57
ERS20602169	EC_IMVM_1132	GCA_964203965	7,48,186	5,185,087	43.3	586	10,756	99.64	0.80
ERS20602185	EC_IMVM_1148	GCA_964204045	1,120,594	1,221,858	275.1	1,883	294	36.37	2.55
ERS20602209	EC_IMVM_1172	GCA_964204105	2,312,343	5,111,657	135.7	344	20,272	99.64	0.22
ERS20602219	EC_IMVM_1182	GCA_964204495	3,541,486	6,290,664	168.9	2,701	608	99.64	47.33
ERS20602230	EC_IMVM_1193	GCA_964204435	12,657,602	8,018,205	473.6	9,972	352	96.95	74.57
ERS20602272	EC_IMVM_1235	GCA_964204415	19,666,377	5,077,533	1,162.0	338	19,989	99.64	0.22
ERS20602293	EC_IMVM_1256	GCA_964204335	16,382,498	4,993,863	984.2	335	16,652	99.64	1.13
ERS20602308	EC_IMVM_1271	GCA_964204395	711,047	5,496,951	38.8	1,609	1,195	99.64	3.32
ERS20602338	EC_IMVM_1301	GCA_964204405	977,154	5,116,193	57.3	412	26,804	99.60	0.45
ERS20602643	EC_IMVM_1609	GCA_964204315	1,424,041	10,238,381	41.7	15,005	251	96.79	125.99
ERS20602730	EC_IMVM_1696	GCA_964204555	14,039,373	5,074,554	830.0	428	27,338	99.48	0.50
ERS20602797	EC_IMVM_1763	GCA_964204485	141,992	4,813,034	8.9	1,595	1,341	98.68	4.77
ERS20602802	EC_IMVM_1769	GCA_964204575	1,113,103	5,054,852	66.1	156	45,867	99.64	0.22
ERS20602844	EC_IMVM_1813	GCA_964204515	5,783,390	5,727,626	302.9	1,581	1,298	99.48	28.06
ERS20602849	EC_IMVM_1818	GCA_964204365	194,378	4,992,564	11.7	1,777	1,393	98.60	3.56
ERS20602858	EC_IMVM_1827	GCA_964203925	199,029	5,114,163	11.7	954	2,759	98.63	1.56
ERS20602859	EC_IMVM_1828	GCA_964204545	19,962,109	5,215,695	1,148.2	310	18,707	99.64	0.49
ERS20602867	EC_IMVM_1837	GCA_964204345	175,658	4,961,381	10.6	1,391	1,701	97.81	3.50
ERS20602869	EC_IMVM_1839	GCA_964204275	688,994	5,182,306	39.9	534	19,218	99.64	0.53
ERS20602871	EC_IMVM_1842	GCA_964204375	6,742,819	4,841,215	417.8	139	39,235	99.64	0.22
ERS20602882	EC_IMVM_1853	GCA_964204305	18,014,741	5,069,526	1,066.1	327	19,104	99.64	0.33
ERS20602883	EC_IMVM_1854	GCA_964204635	1,461,638	4,955,554	88.5	281	28,761	99.64	0.22
ERS20602885	EC_IMVM_1856	GCA_964204505	272,482	4,945,019	16.5	340	19,907	99.64	0.25
ERS20602886	EC_IMVM_1857	GCA_964204285	392,686	4,954,918	23.8	345	23,389	99.64	0.22
ERS20602887	EC_IMVM_1858	GCA_964204455	13,406,208	4,955,092	811.7	263	27,625	99.64	0.22
ERS20602913	EC_IMVM_1884	GCA_964204465	879,051	5,227,669	50.4	613	11,260	99.64	0.65
ERS20602918	EC_IMVM_1889	GCA_964204475	2,734,584	4,521,863	181.4	337	13,823	99.64	0.10
ERS20602941	EC_IMVM_1912	GCA_964204385	7,815,271	9,532,475	246.0	7,845	286	98.68	99.33
ERS20602950	EC_IMVM_1921	GCA_964204595	2,389,334	5,293,642	135.4	1,485	1,568	99.02	15.58
ERS20602953	EC_IMVM_1924	GCA_964204295	8,131,718	10,548,406	231.3	21,596	164	96.48	97.54
ERS20602959	EC_IMVM_1930	GCA_964204615	122,574	4,371,274	8.4	6,189	367	84.21	18.73
ERS20602967	EC_IMVM_1938	GCA_964204565	2,164,633	8,657,258	75.0	13,722	215	95.44	75.91
ERS20602978	EC_IMVM_1949	GCA_964204585	5,514,916	4,969,346	332.9	354	13,898	99.25	0.18
ERS20602989	EC_IMVM_1960	GCA_964204015	161,736	5,007,930	9.7	1,221	2,280	99.06	3.12

## Data Availability

Genome assembly accession numbers are listed in Table 1. The human-filtered metagenomic sequencing data used for assemblies are available in the European Nucleotide Archive (ENA) repository: project accession no. PRJEB77434 and file accessions ERS20601047-ERS20603005. All scripts and Snakemake pipelines are publicly available on Github: https://github.com/motleystate/moonshot.git. The commit hash corresponding to the code version used in this study is 80a68a0d164e3ee07e291b885673fc28f747114a.
